# Supplementary material for: Acyl Hydrazides and Acyl Hydrazones as High-Performance Chemical Exchange Saturation Transfer MRI Contrast Agents
Source: Pharmaceuticals (Basel). 2023 Apr 23;16(5):639. doi: 10.3390/ph16050639 (PMC10223748; doi:10.3390/ph16050639)
Supplement: Supplementary file 1 [file pharmaceuticals-16-00639-s001.zip › pharmaceuticals-2301065-supplementary.pdf]

Supplementary Materials

Acyl hydrazides and acyl hydrazones as high performance chemical exchange saturation transfer MRI contrast agents

Shaowei Bo[1, †], Dong Zhang[2, †], Mengjie Ma[2], Xukai Mo[2], Julia Stabinska[3,4], Michael T. McMahon[3,4,*], Changzheng Shi[2,*], Liangping Luo[1,2,*]

1 Department of Medical Imaging, The Affiliated Guangdong Second Provincial General Hospital of Jinan University, Guangzhou 510317, China.
2 Medical Imaging Center, The First Affiliated Hospital of Jinan University, Guangzhou 510632, China.
3 The Russell H. Morgan Department of Radiology and Radiological Science, The Johns Hopkins University School of Medicine, Baltimore, MD 21287, USA.
4 F.M. Kirby Research Center for Functional Brain Imaging, Kennedy Krieger Institute, Baltimore, MD 21287, USA
* Correspondence: tsczcn@jnu.edu.cn(C.S.); mcmahon@mri.jhu.edu(M.T.M.); tluolp@jnu.edu.cn(L.L.)
† These authors contributed equally to this work.

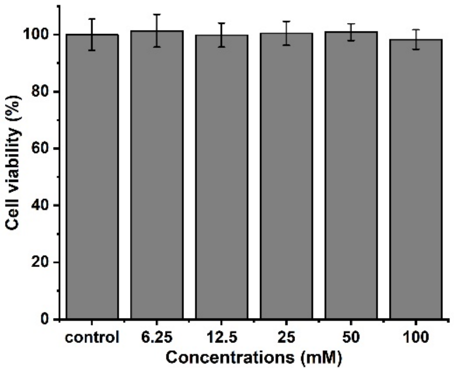

Figure S1. Cytotoxicity assay of **ADH** on 4T1 cells with different concentration range.

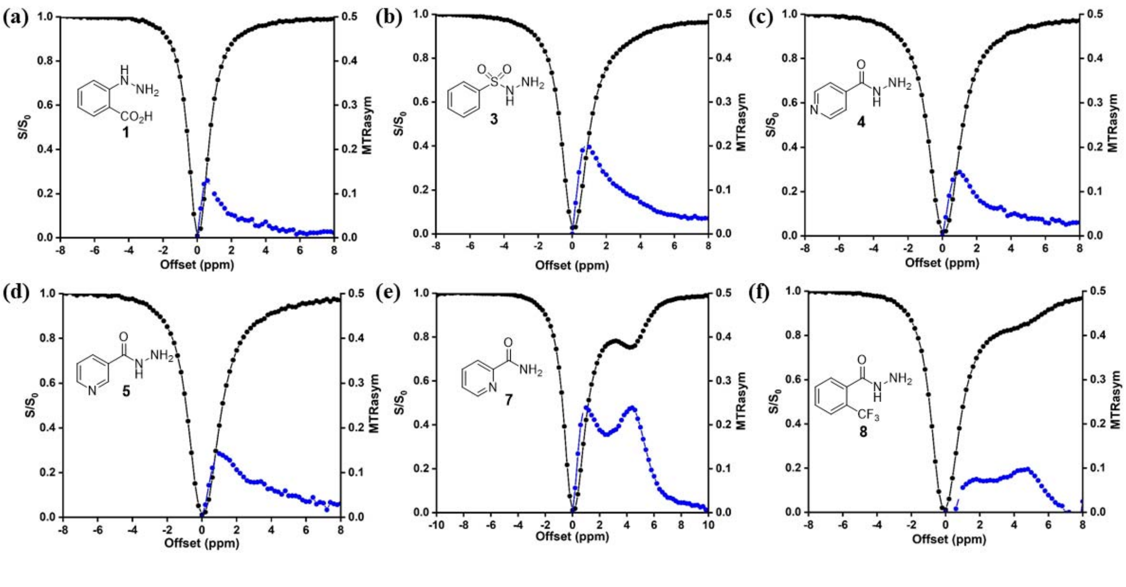

Figure S2. Z spectra and MTR_{asym} spectra of acyl hydrazide **1** (a), **3** (b), **4** (c), **5** (d), **7** (e) and **8** (f). Experimental conditions: CEST data were obtained at 40 mM concentration; pH 7.2, tsat = 3 s, B_1 = 3.6 μT, 37 °C.

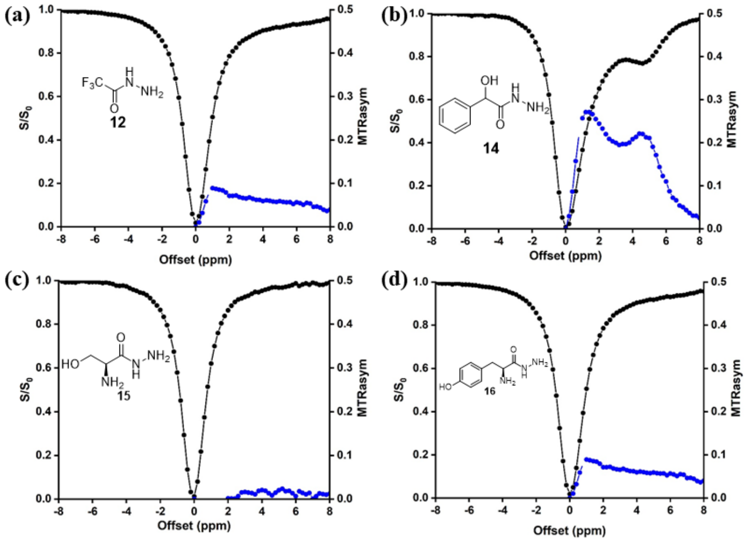

Figure S3. Z spectra and MTR_{asym} of acyl hydrazide **12** (a), **14** (b), 1**5** (c), and 1**6** (d). Experimental conditions: CEST data were obtained at 40 mM concentration; pH 7.2, tsat = 3 s, B_1 = 3.6 μT, 37 °C.

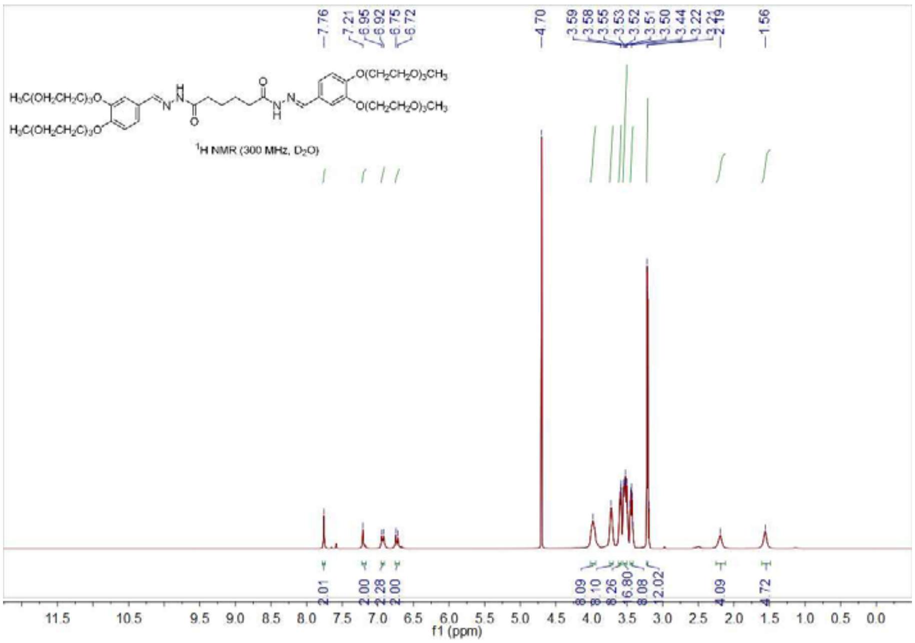

Figure S4. ^{1}H NMR of acyl hydrazone **17**

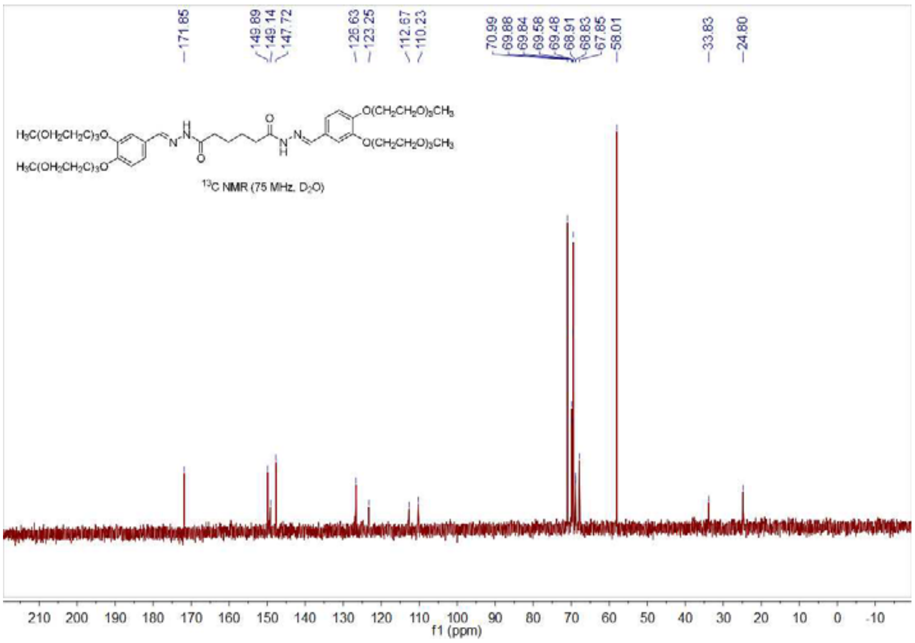

Figure S5. ^{13}C NMR of acyl hydrazone **17**

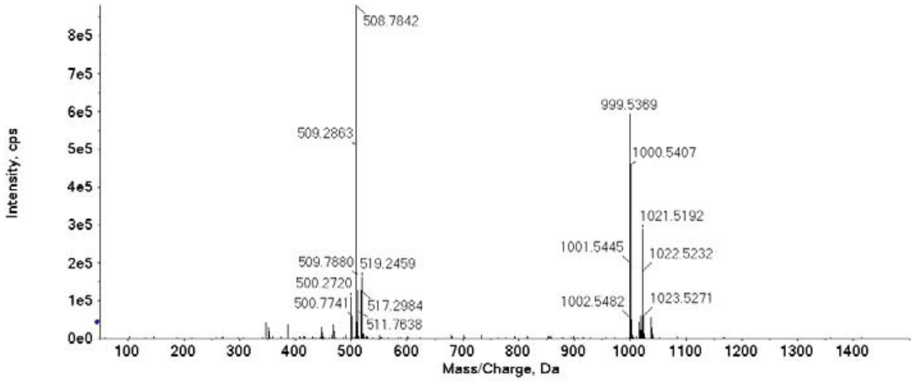

Figure S6. HRMS of acyl hydrazone **17**, calcd for $C_{48}H_{79}N_4O_{18}^+$ ([M+H]$^+$) 999.5384, found 999.5369.
